# A Systematic Review of In-Vehicle Physiological Indices and Sensor Technology for Driver Mental Workload Monitoring

**DOI:** 10.3390/s23042214

**Published:** 2023-02-16

**Authors:** Ashwini Kanakapura Sriranga, Qian Lu, Stewart Birrell

**Affiliations:** Institute for Clean Growth and Future Mobility, Coventry University, Coventry CV1 5FB, UK

**Keywords:** HR, HRV, respiration, mental workload, conditional automation

## Abstract

The concept of vehicle automation ceases to seem futuristic with the current advancement of the automotive industry. With the introduction of conditional automated vehicles, drivers are no longer expected to focus only on driving activities but are still required to stay alert to resume control. However, fluctuations in driving demands are known to alter the driver’s mental workload (MWL), which might affect the driver’s vehicle take-over capabilities. Driver mental workload can be specified as the driver’s capacity for information processing for task performance. This paper summarizes the literature that relates to analysing driver mental workload through various in-vehicle physiological sensors focusing on cardiovascular and respiratory measures. The review highlights the type of study, hardware, method of analysis, test variable, and results of studies that have used physiological indices for MWL analysis in the automotive context.

## 1. Introduction

**Figure 1:**
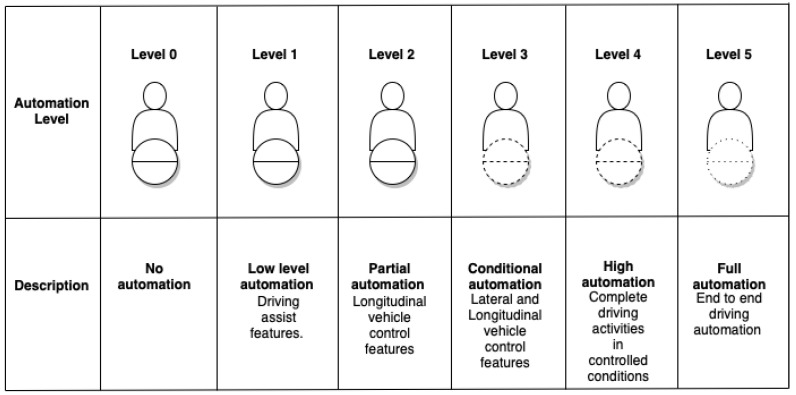
An illustration of SAE levels of automation.

As the race to implement full driving automation continues around the globe, automotive applications have advanced tremendously in the past decade. A standard called the SAE J3016 [1] that represents six levels of vehicle automation, as represented in Figure 1, has been introduced by the Society of Automotive Engineers (SAE) to facilitate and compare changes and advancements in the automotive domain. The system enables automakers and suppliers to correlate each level of automation to user experiences. Level 0 in the hierarchy represents vehicles that are manually controlled. Drivers are in complete control of the vehicle except for some emergency safety features. Vehicles that fall under the category of Level 1 automation possess the lowest level of automation, with features such as adaptive cruise control and steering assist. Level 2 vehicles are often referred to as partially automated, where the vehicles can control acceleration, braking, and the steering controls. A crucial change occurs between Level 2 and Level 3 vehicles, where the concept of conditional automation is introduced. Conditionally automated vehicles can handle lateral and longitudinal vehicular decisions and make use of artificial intelligence for decision making, enabling drivers to engage in non-driving activities. However, conditionally automated vehicles still require a driver to take over the vehicular controls in emergencies. Vehicles that fall under the category of Level 4 do not require any human intervention for vehicular operations. The operation of Level 4 vehicles might be limited, as they will be affected by external factors such as weather conditions. The topmost position in the automation hierarchy is occupied by Level 5 vehicles, capable of intelligent decision making and self-driving in all weather conditions. Human intervention in Level 5 vehicles will be at its minimum, where interaction is only expected at the start and at the final destination.

Among the levels of automation, the major change occurs between Level 2 and Level 3, where drivers are allowed to engage in non-driving-related tasks (NDRT). Vehicles at this level still require their drivers’ attention and intervention when the automation has reached its limits or in instances that require manual manoeuvring. In such situations, a Take Over Request (TOR) in the form of alerts/notifications is presented to drivers through audio or visual means. TORs require drivers to be prepared to resume control of vehicles, often demanding a sudden shift from a non-driving state to a driving state. The vehicle take-over process is a multistep process that includes perception of the TOR, cognitive processing, and action selection [2]. The process might be affected by several external and internal factors, as drivers may be involved in an NDRT or might be physically or mentally incapable of acquiring control immediately and suffer from an “out of loop” situation [3]. The degree of disconnect highly depends on the complexity of the driving situation during the take overs and the level of driving experience of the individual. De Waard [4] has elaborated on three levels of driving: the strategic level (route decisions), the manoeuvring level (behaviour/reaction to external traffic conditions), and the control level (basic vehicular control operations). Driving performance can be assessed on similar grounds, where driving demands can vary at different driving levels. Performance variations can be caused by factors and workload in the interior and exterior vehicle environments, where personal limitations and driver mental workload become important factors.

Mental workload (MWL), sometimes referred to as cognitive workload, is a dynamic concept that acquires different meanings depending on the outcome desired. Stanton [5] describes MWL as a multidimensional concept that is usually defined by the characteristics of the tasks presented and the individuals performing them. However, there are two main components of MWL: the task demands and the impact they have on the individual performing the task. Further, on an operational level, Stanton has defined the MWL to be the level of attention required to meet the objective and subjective criteria of the demands. In the automotive context, drivers are often presented with multiple tasks that need to be performed simultaneously, and the MWL can be the impact of the driving and non-driving activities on the driver [6]. It can be concluded that the term “mental workload”, in the vehicle context, can be typically defined in terms of “task capacity” while performing a combination of simple and complex vehicular tasks. In recent years, the concept of MWL has been researched in several domains to understand the performance limits of humans. For instance, researchers have found that teleoperation tasks cause a trend of an increased MWL that is usually affected by subjective factors [7]. The increase in the MWL can potentially lead to errors, leading to failure in task completion. Humans working in complex environments, especially in the healthcare domain, require their cognitive resources to be allocated to several important tasks. A cognitive overload for individuals working in the healthcare domain can impact their performance and cause serious, life-threatening accidents, especially in surgical environments. For such reasons, researchers have focused on detecting and assessing the MWLs of healthcare workers through various measures [8,9,10]. Similarly, the effect of multiple task handling on performance, as reflected by MWL, has also been studied in air traffic controllers [11], electric overhead traveling crane operators [12], and human system interface operators in nuclear power plants [13]. In the consumer context, MWL has been studied to understand the Quality of Experience (QoE) and User Experience (UX) [14].

The measurement of MWL is as flexible as its theoretical definition, depending on the domain and context. In the literature, MWL measurement methods are broadly classified into two categories: subjective methods and objective methods. As MWL is a multimodal concept, researchers usually select a battery of measures that includes subjective and objective methods that tap different dimensions of the MWL. Subjective scales consider the experience of the individual performing the specific task. The data for subjective scales are collected from individuals in the form of questionnaires, which makes the procedure low-cost and easy to implement. Some of the most widely used subjective scales for MWL measurement are the Cooper–Harper scale [15], the Subjective Workload Assessment Technique (SWAT) [16], and the NASA Task Load Index (TLX) scale [17]. Subjective scales are further categorised into unidimensional or multidimensional scales, a difference that affects their sensitivity in measuring MWL. The Cooper–Harper scale is a unidimensional decision-tree based scale that is widely used in the aviation industry for assessing aircraft flying quality. The information processing capabilities of humans are usually complex and need to be analysed at different levels, considering task demand and different cognitive modalities [18]. Hence, multidimensional scales are known to be more sensitive and accurate. The SWAT technique uses a three-dimensional subjective scale emphasising time on road, mental effort load, and psychological stress load. Similarly, the NASA-TLX scale uses a six-dimensional scale: mental demand, physical demand, temporal demand, performance, effort, and frustration level.

The NASA-TLX scale has been implemented and tested in several domains, proving to be more sensitive when compared to other subjective scales for measuring MWL [19]. In the automotive domain, the DALI (Driving Activity Load Index) [20] has been developed as a revised version of the NASA-TLX to make it more suitable for driving activities. The basic principle of DALI mimics the NASA-TLX, with six pre-defined factors, and only varies in the choice of the factors involved in defining the workload score. The scale has been used in several automotive studies and has proven to be advantageous for identifying the origin of the driver’s MWL [21,22,23]. Although subjective measures are simple and easy to implement, the assumption is that humans are capable of recognising increases in workload demands. Further, subjective scales also assume that increases in stress levels increase the MWL, regardless of all the other indications [18]. Subjective scales are often not suitable for commercial and real-time MWL analysis as they are considered pre-task and post-task self-reported assessments and commonly suffer from post-task bias during long duration tasks. Real-time MWL analysis requires objective analytical methods that can indicate continuous changes throughout the task.

Driving performance measurements employing primary and secondary tasks are a popular objective approach to assessing the driving MWL. Some of the commonly observed vehicular parameters are speed, steering movement, and the accelerator and brake pedal positions. Researchers have used visual, audio, and haptic secondary tasks to observe the variation in the primary driving task, which can be used as an indicator to assess the MWL [24,25,26,27,28]. Physiological indicators of MWL have gained an immense amount of attention in several domains, considering their objective nature. The response of the human body to external sources of workloads can be effectively observed through physiological signal markers that are not heavily affected by subjective opinions. Overall, it can be considered an indirect measure that can be related to MWL and has a relatively quicker response to sudden shifts [29]. Physiological processes that include heart activity, respiration, digestion, and sexual arousal are involuntarily regulated by the peripheral component of the autonomic nervous system. The autonomic nervous system has three distinct divisions, namely, the sympathetic (SNS), parasympathetic (PNS), and enteric. The activation of the SNS and PNS can be directly observed in HR and HRV variations. Some commonly used SNS- and PNS-related physiological indicators are heart rate (HR) [30], heartrate variability (HRV) [31], respiratory rate (RR) [32], galvanic skin response (GSR) [33], and electrodermal activity (EDA) [34]. Eye-related data such as pupil size are also a result of autonomic activity and have been an important component of MWL research, as visual and mental tasks are highly correlated [35]. Previous research has suggested that an increase in the cognitive demand or an increase in the MWL can result in increased blood flow in the frontal cortex of the brain. Several studies have used techniques such as functional near infrared spectroscopy (fNIRS) [36,37], an optical technique that facilitates the study of hemodynamic oscillations in the cortical regions. The sample entropy of fNIRS signals is particularly associated with mental workload [38]. Other optical techniques, such as electroencephalography (EEG) [39], have also been employed to study MWL in the automotive context. As mentioned, MWL is a multidimensional concept where researchers have generally considered a combination of subjective and objective methods. In this paper, we conducted a systematic review of the objective physiological approaches using cardiovascular and respiratory physiological indicators (HR, HRV, and RR) to measure driver MWL in driving scenarios, the in-cabin sensors used for physiological signal retrieval, and the data analysis methods, which is a novel addition compared to previous reviews. Additionally, a short survey of other physiological indicators that are included in the selected papers and their impact on MWL has been presented. The review provides a comprehensive understanding of the physiological perspective on mental workload that benefits human-factors-related automotive research.

## 2. Physiological Mental Workload Indicators

### 2.1. Cardiovascular Measures

Heart rate (HR) is a cardiac activity metric that corresponds to the number of heartbeats per unit time (per minute). HR is commonly derived from the electrocardiogram (ECG) signal, which represents the electrical activity of the heart. HR can also be retrieved from photoplethysmography (PPG) devices that use light and photodetectors to observe volumetric changes of the blood through human skin. In the task of mental workload assessment, the topic of interest would be the variation in the interval between successive heartbeats. Heartrate variability (HRV) is a term that generally refers to changes in the inter-beat interval (IBI). The heart is known to be supplied with nerves from the sympathetic and the parasympathetic branches of the autonomic nervous system (ANS). The two systems show opposing effects on the HR, where activation of sympathetic system increases the HR and activation of parasympathetic system decreases the HR [40]. Several initial studies that were been conducted as early as in the 1980s have concluded that changes in the MWL can be observed through changes in the cardiovascular responses [30].

#### Heartrate Variability (HRV)

The traditional method for measuring mental workload using HRV metrics uses the time domain and frequency domain components [41,42]. The time domain category comprises statistical and geometrical measures that determine the time variability between heartbeats. Time domain analysis through statistical methods is further classified into two categories: (a) Direct measurements of Normal-to-Normal (NN) intervals, and (b) Differences between NN intervals. The NN intervals can also be derived as a geometrical pattern that can be studied using different approaches: (1) basic measurement of the pattern converted into a HRV measure, (2) interpolation of the geometrical pattern, and (3) categorisation of the geometrical pattern into classes of HRV. The analysis of HRV in the frequency domain usually involves the study of the power spectral density (PSD), which provides information about how power distributes as a function of frequency in the HRV signal. The spectral analysis of HRV is based on the Fourier theory, where a signal can be represented as the sum of sinusoidal signals consisting of amplitude, phase, and frequency components. The literature has confirmed that the spectral components of HRV can be categorised into very low frequency (VLF), low frequency (LF), high frequency (HF), and ultra-low frequency (ULF) components in short-term and long-term readings [43]. Short-term readings generally consider 2–5 min of HRV data, whereas long-term readings consider HRV data up to 24 h. The time and frequency domain components of HRV are summarised in Table 1.

### 2.2. Respiratory Measures

The biggest oscillator in the human body that is involved in the maintenance of homeostasis is the phenomenon of respiration. It is well known that respiratory activation additionally influences psychological and behavioural processes along with metabolic changes [44]. RR also exhibits a close relationship with HR, as the coupling between them can provide information to be used as an index to study the vagal control of the heart [45]. Several studies have indicated that the respiratory rate (RR) is affected by emotional and cognitive demands reflecting limbic and paralimbic influences [46,47]. The psychophysiology effects of respiration are generally studied based on measures related to time, volume, and gas exchange [48,49]. The components and features of the RR are generally analysed using time, volume, and spectral parameters. The most popularly used features are the respiratory rate, inspiratory time, expiratory time, timing ratio, tidal volume, minute ventilation, and spectral power. A summary of RR features has been presented in Table 2.

### 2.3. Other Physiological Measures

According to the literature, mental workload (MWL) and driver distraction are often related to each other, such that the same secondary task used to measure distraction can be used to measure MWL [50]. As the trend towards in-vehicle infotainment systems has modernised vehicles, distractions are often induced visually or cognitively [51]. Eye-related measures such as blink frequency, blink duration, and pupil dilation are recorded using head-mounted gear that cannot be used outside experimental conditions, as it causes discomfort. Further, if one opts for optical instruments such as cameras to gather eye-related data, multiple installations may be required, considering head movement and occluded situations. EDA (Electrodermal Activity) is a collective term used to define the bio-electrical changes that occur in the skin, which is one of the most useful indices of the sympathetic activity impacting sweat gland activity.

The functional concepts of EDA closely relate to psychophysiological activity, which makes it an important objective measure for understanding arousal, attention, and emotional responses. EDA is classified into two categories: skin conductance level (SCL) and skin conductance responses (SCR). SCR data are obtained through the placement of electrodes in positions such as below the distal phalanx of the index and middle finger. Similarly to with eye-related measures, the signal acquisition of the EDA components might become problematic, as data are generally affected by noise artifacts induced by driving movement.

## 3. Methods—Search Methods and Eligibility Criteria

In this review, we aimed to select research that mainly included cardiovascular and respiratory physiological signals. As respiration and heartrate cause significant oscillations in the human body, the current gap in the research indicates that the signals can be obtained in a non-invasive and contactless manner using several advancing technologies. Signals such as an ECG require human skin contact, whereas the majority of the eye-related measures are reliably collected through head-mounted gear. Hence, the review would provide proof of the relevancy of cardiovascular and respiratory measures, individually, in the context of MWL.

**Figure 2:**
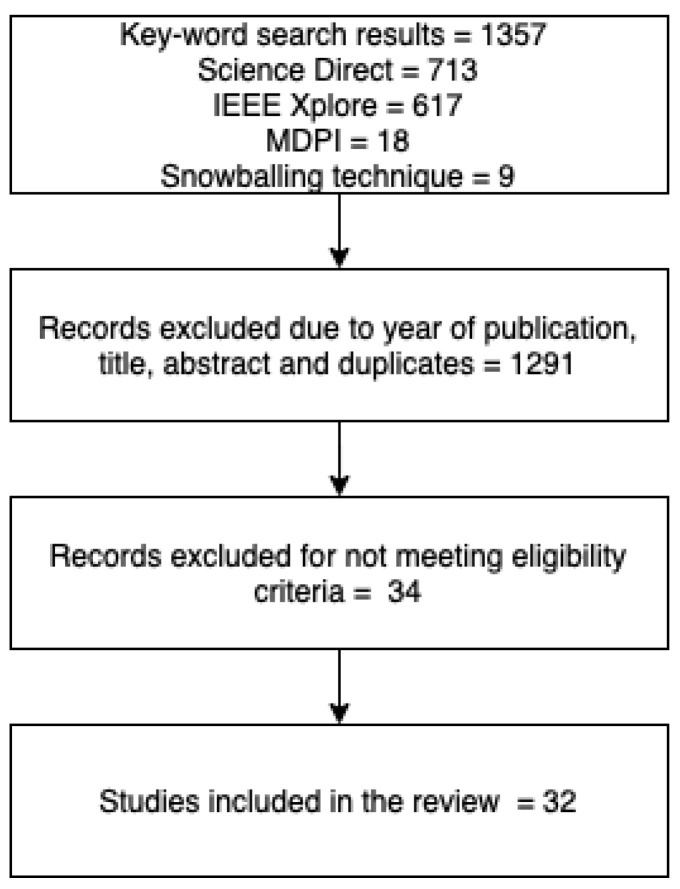
Flowchart of the selection process for the systematic review.

In this paper, we aim to only include original research where primary data were collected and analysed from the year 2015 onwards. MWL-related studies in the domain car driving, only, have been included, while studies related to MWL in other means of transport, i.e., aircrafts, trains, bicycles, and bikes, were excluded. Studies that were not conducted in simulated or on-road driving environments were excluded. The selection process for the review was in accordance with PRISMA guidelines and has been depicted in the Figure 2. The data extracted from the selected papers were limited to the type of study, hardware used, test variable against which MWL was measured, and type of validation. The type of study indicated if data were collected in a simulated environment or in a real driving scenario. The data collection hardware, i.e., the types of sensors used to record physiological data, has been extracted from the selected papers. Finally, the test variable against which the MWL was measured and the validation methods were extracted. We accessed Science Direct (Elsevier’s platform for accessing peer reviewed literature) as the main database, but also explored papers from the IEEE Xplore and MDPI databases to access papers via institutional sign in. We also employed the snowball rolling technique to identify relevant studies from the identified papers. The key words used for the search are: “driver” “mental workload” “workload” “cognitive workload” “HR” “Heart rate” “HRV” “Heartrate Variability” “RR” “Respiratory Rate”.

## 4. Results

### 4.1. Type of Study

Among the 32 papers selected, 11 studies were conducted in real driving scenarios, whereas 21 studies were conducted in a driving simulator. Although on-road driving data provide more naturalistic information, it is difficult to maintain uniformity considering the weather conditions and the time of day [52,53]. Similarly, certain tasks can distract from driving activities, leading to accidents. On-road based studies require carefully examined task parameters and test variables that may require additional assistance [54]. Simulator studies have proven to be as reliable as on-road studies, with an added advantage of safety. However, the risk of simulator motion sickness and light-headedness may affect several participants. Several studies have a percentage of participants who reported discomfort or were excluded from the research due to motion sickness [55,56,57].

### 4.2. Hardware

**Figure 3:**
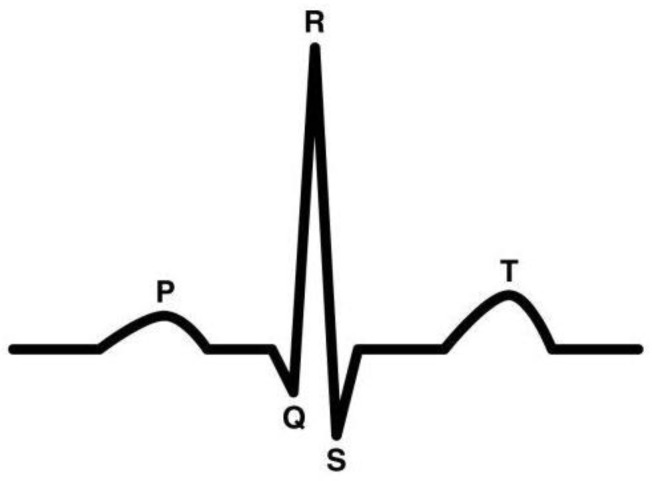
QRS complex of an ECG curve.

The studies included in this review have measured cardiovascular parameters using different types of sensors. Electrode-based ECG has been the most frequently used data collection hardware for HR and HRV data [21,30,52,54,56,58,59,60,61,62,63,64,65,66,67,68,69,70,71,72,73]. HR and HRV analysis typically requires three leads in the ECG to detect the R peak in the QRS complex [74]. The QRS complex is the graphical representation that corresponds to the contraction of large ventricular muscles and the depolarization of right and left ventricles of the heart. The Q wave deflects downwards followed by the R peak which is the dominant peak in the ECG signal. The R peak is followed by the S wave that deflects downwards, similarly to the Q wave. A representation of the QRS complex is depicted in Figure 3. Although ECG is one of the most used and reliable sources for studying HR and HRV, it is susceptible to different types of noises that can even be a result of the wrong electrode placement. In comparison to the electrode-based ECG method, wearable chest straps to retrieve HR data have also been used [53,55,57,75,76,77,78] and have been proven to be an easier implementation. HR data have also been retrieved using photoplethysmography (PPG), a method that detects blood volume changes in cardiac cycles through light-emitting diodes (LEDs) [79,80,81,82]. PPG-based sensors are considered non-invasive in comparison to traditional ECG sensors but are prone to noise and data loss due to motion artifacts. The studies that included RR as a metric have used chest respiratory bands for signal retrieval [30,56,58,59,61,61,70,71]. Overall, ECG and chest respiratory bands have been the most frequently used apparatuses for recording cardiovascular physiological data.

### 4.3. Test Variable

The reviewed studies have taken multiple approaches to inducing a MWL in drivers. The methods can be categorised into in-vehicle, external, and driving-task-related variables. The in-vehicle variables include interaction with passengers [79,82] and the performance of secondary tasks through cognitive visual and auditory inputs. Secondary tasks can be a wide variety of tasks ranging from interacting with in-vehicle infotainment systems [57,68], performing n-back tasks [57,62,71,72], and listening to music [65,77] to verbal learning and arithmetic tasks [30,69]. The external variables include road, traffic intensity [6,59,68,73,79,82] and weather-related [68,82] metrics that have been tested to induce MWL. The road-related variables include comparisons between rural, urban, motorway, and tunnel roads [52,53,68,82] and road-geometry-related metrics [67,81]. Driving-related tasks deal mostly with understanding the behaviour of the driver and their MWL in response to certain changes in vehicle manoeuvres, such as lane changing, overtaking, and behaviour at intersections [55,56,58,60,61,64]. Overall, variation in the MWL has been studied for specific external situations or to test the effect of new vehicle technologies. The variables have been selected and designed according to the overall aim of the respective study, indicating that there are no dedicated tasks that promise to change MWL in drivers in every condition.

### 4.4. Data Analysis

Studies designed to detect and analyse MWL are generally factorial designs where the experimental conditions can be classified into several levels corresponding to one or more affecting factors. When such scenarios are tested across a group of participants or several clusters of participants, statistical analysis techniques have been used to detect significant differences. Analysis of Variance (ANOVA) is a common method employed by majority of studies selected in this review. Several variants of the ANOVA such as multivariate [61,71,72,73,76,79,81], one-way [57,78], two-way [30,67], repeated measures [54,55,64,75] or mixed analysis [65,77] have been employed according to the selected features. Post-hoc pairwise comparison between two measures or two groups of participants have been employed by several studies for further analysis [56,64,71,72,76]. Some of the other statistical analysis methodologies used are Pearson correlation [59], parametric analysis with T-scores [68], Mann-Whitney U tests [66], and log-likelihood [80].

The use of machine learning (ML) for data analysis is becoming a popular alternative for standard statistical analysis. Supervised algorithms such as decision trees, discriminant analysis, logistic regression, support vector machines, nearest neighbour, and ensemble classifiers have been trained using labelled data obtained from subjective and task completion results. Similarly, unsupervised algorithms such as Random Forest, CNN (Convolutional Neural Network), LSTM (Long Short-Term Memory), K-Nearest Neighbour (K-NN), and XGBoost have been used on physiological data for classification. In such cases, the results of statistical analysis of subjective measures have been used as a method of validation. Machine learning classification algorithms have proved to be very efficient rendering classification results that are at least above 90% accurate in most cases.

### 4.5. Findings

#### 4.5.1. Cardiovascular and Respiratory Components

- Variation in the MWL is known to be reflected in the variations in the HR and HRV parameters, which is confirmed by some of the studies in this review.
- The impact of RR has been minimal when it has been considered as a feature in studies employing machine learning [58,71], but individually it seems insignificant due to many external factors such as speech and fatigue affecting it [30,61].
- Studies that used secondary tasks as a test variable against the MWL showed significant changes in the HR and HRV features with an increase in the difficulty of performing driving or non-driving tasks [30,55,57,61,62,69,72,75].
- Melnicuk et al. [57], Tjolleng et al. [62], Biondi et al. [75], and Hidalgo et al. [30] found the time domain metrics of HR and HRV to be more sensitive to changes in the MWL. On the contrary, Heine et al. [72], Tozman et al. [55], and Cardone et al. [69] observed no significant results from time domain measures, but concluded that the low frequency and high frequency components of HRV were better indicators.
- Two studies used music to analyse the effects of auditory input, and driving impairment was observed in choleric drivers in one study [65], while the other concluded that there were no significant changes [77].

When external parameters such as traffic intensity, comparisons between road types, and weather conditions were used as test variables, the outcomes seemed to vary. Sugiono et al. [52] observed the least mean R-R interval on motorways, indicating an increase in HR. Similarly, Perello-March et al. [68] and Tavakoli et al. [82] also observed an increase in HR on motorways compared to urban roads. Increases in traffic intensity had a similar impact, and Schmidt et al. [59] have found a high correlation between an increase in the HRV and subjective feedback. However, Melnicuk [76] indicated the time domain R-R-interval-based parameter RMSSD to be insignificant between motorways and rural roads. Individual differences among participants tackling traffic intensity were emphasised by Tavakoli et al. in their study [80]. Road geometry and driving through optical and non-optical tunnels did not show any significant MWL differences according to studies conducted by Jacob et al. [81], Shao et al. [67], and Luo et al. [53]. Driving-behaviours-based variables such as lane keeping, overtaking, and behaviour at intersections seem to have a minimal impact on MWL [60,63,64]. The detailed review of the results is formulated in Table 3.

#### 4.5.2. Other Physiological Measures

The impact of other physiological signals on MWL have been derived from the selected papers as an incomprehensive review as compared to the systematic focus on cardiovascular and respiratory signals in the previous section. Some studies in the review have concluded that eye-related features such as blink frequency and blink duration show significant variation in response to an externally induced MWL such as that caused by secondary tasks or traffic density [52]. In contrast, some studies also report no significant changes in blink behaviour in induced MWL conditions [62]. Several studies in this review have included components of EDA as one among the several objective measures [51,52,57,61,63,64,66]. The results obtained by Meteier et al. [70] argued that a combination of EDA and RR signals can be used to detected verbally induced MWL. Similarly, MWL was induced by secondary tasks such as the N-back task, where a higher SCR was observed for the 2-back task condition and across different scenarios [68]. SCL values seemed to notably differ in the small 30 s window of time after task completion in the study conducted by Loeches et al. [73]. The study further indicated that SCL is more sensitive to short-term physiological variations due to MWL compared to other signals. There were no significant results when the MWL was induced based on lane keeping styles, as indicated by Kuo et al. [64]. It is observed that the MWL has a significant impact on SCR components and that they can be used as a measure in combination with the other physiological signals.

## 5. Discussion and Future Scope

MWL (Mental workload) is an important concept that facilitates a human-centric approach to advancements in vehicle automation. Although MWL is a dynamic concept, it can be perceived as the drivers’ task capacity or as the impact of driving and non-driving activities on drivers in the automotive domain. In this review, we reviewed the impact of cardiovascular parameters such as HR (Heart Rate), HRV (Heart rate Variability), and RR (Respiratory Rate) on the MWL in vehicular scenarios. The conclusions from the components of the review are as summarised below:

1. Type of study: It was observed that on-road studies have mostly focused on the MWL induced by external and driving-style-based variables. Most simulator-based studies have focused on the MWL induced by secondary driving tasks. We conclude that simulator studies are ideal for studying MWL in Level 3 vehicles, as the emphasis is on take-over behaviour that might be dangerous to implement using on-road experiments. Additionally, simulator studies provide ease in the design of scenarios with variable weather and traffic conditions, proving to be beneficial.
2. Hardware: Most of the studies have used a traditional electrode-based ECG (Electrocardiogram) for HR and HRV data acquisition. Some studies have used PPG (Photoplethysmography) based sensor chest bands for RR data and smartwatches as an alternative to an ECG. Data acquisition during driving activities is a tedious task that requires precision and causes discomfort in participants due to the use of body mounted sensors. We conclude that a research gap exists in the field of contactless physiological monitoring in vehicular environments that needs to be addressed. Currently, advancements in the applications of short-range radars have been extended to cardiovascular and respiratory physiological sensing that can be employed for data acquisition purposes in the automotive context.
3. Test variable: In the review, it was observed that experimental scenarios and test variables were selected according to the overall aim of the study. MWL can be induced in multiple ways that include variables relating to road infrastructure, specific driving behaviour, and secondary visual and auditory tasks. In the case of Level 3 vehicles, drivers are allowed to be engaged in non-driving activities when the vehicle handles lateral and longitudinal driving decisions. Hence, a MWL induced by a combination of in-vehicle secondary activities can be beneficial in understanding take-over behaviour.
4. Data analysis: Most of the reviewed studies rely on statistically proven variations in physiological data to infer changes in the MWL. However, most of the statistical methods employed only account for highly significant changes in physiological signals and essentially eliminate any moderate and low-level changes that are insignificant but are present. The use of supervised and unsupervised machine learning (ML) algorithms for MWL has proven to be an efficient solution, by means of which most of the studies have achieved over 90% accuracy. Algorithms such as CNN (Convolutional Neural Networks), LSTM (Long Short-Term Memory), Random Forest, and K-Nearest Neighbour are known to excel at pattern recognition problems. Hence, we conclude that ML algorithms are the ideal choice for data analysis for MWL detection and classification.

Future studies that investigate MWL using HR, HRV, and RR could use simulator environments, employing contactless and non-invasive data acquisition methods such as short-range radars with specific test variables and ML data analysis tools for more efficient results in the case of Level 3 vehicles. The test variables could be changed while the methodology is preserved in the testing of various driving and non-driving features in highly automated vehicles to extend the scope of our understanding of MWL. The combination of ML algorithms and contactless physiological monitoring can also be extended to enhance vehicle safety features or for the addition of emergency services.

## 6. Conclusions

The impact of cardiovascular and respiratory physiological parameters, namely heartrate (HR), heartrate variability (HRV), and respiratory rate (RR), on the mental workload (MWL) of drivers has been addressed in this review. Mixed results indicating the significance of HR and HRV in MWL variation were observed throughout the review. Many studies confirmed that MWL variation is reflected through HR and HRV fluctuations, whereas some studies presented results that were contradictory. One reason for the contradictory results may be the difference in the test variables and the experimental setups. It is important to understand that driving experience and temperament vary from person to person and might affect their physiological activity. Lastly, errors in data acquisition hardware or procedure might also lead to drastic differences in the data analysis procedure by adding or eliminating important patterns and information. RR showed little or no significance in most of the studies as it is heavily affected by speech and other external factors. Although it can be included in the pool of physiological parameters, RR has not been a popular choice for studying the driving MWL. MWL is a multimodal concept and cannot be classified using objective measures alone. We conclude that a combination of subjective and objective data along with the precision of machine learning (ML) algorithms may provide satisfactory results.

## Figures and Tables

**Table 1:** Time and frequency domain components of HRV.

Variable Name (Time Domain)	Description
SDNN	Standard deviation of normal-to-normal intervals.
RMSSD	Square root of the sum of the squares of difference between adjacent normal-to-normal intervals.
SDSD	Standard deviation of differences between adjacent normal-to-normal intervals.
NN50count	Number of pairs of adjacent normal-to-normal intervals differing by more than 50 ms in the entire recording.
pNN50	NN50 count divided by the total number of normal-to-normal intervals.
**Variable name (Frequency domain)**	**Description**
Very low frequency—Short-term	Power in very low frequency range (≤0.4 Hz).
Low frequency—Short-term/Long-term	Power in low frequency range (0.04–0.15 Hz).
High frequency—Short-term/Long-term	Power in high frequency range (0.15–0.4 Hz).
Ultra-low frequency—Long-term	Power in ultra-low frequency range (≤0.003 Hz).
Very low frequency—Long-term	Power in very low frequency range (0.003–0.4 Hz).

**Table 2:** Time and frequency domain components of RR.

Variable Name (Time Domain)	Description
Respiratory rate	Number of breaths per minute.
Inspiratory time	Time taken for inhalation.
Expiratory time	Time taken for exhalation.
Minute ventilation	Product of respiratory rate and tidal volume.
Timing ratio	Ratio of inspiration time to expiration time.
Tidal volume	Amount of inhaled air with each breath.
**Variable name (Frequency domain)**	**Description**
Mid-band spectral power	Spectral power (0.07–0.14 Hz).
High-band spectral power	Spectral power (0.15–0.50 Hz).

**Table 3:** Brief review of the selected 32 papers.

Ref	Parameter	Type of Study	Hardware	Test Variable	Data Analysis	Findings
[54]	HR, HRV	Simulator	Chest belt HR/RR monitor (BioHarness 3, Zephyr technology)	Driving task difficulty	One-factorial repeated measures ANOVA	Increase in task difficulty caused decrease in LF-HRV, decrease in HF-HRV.
[58]	HR, HRV, RR	Simulator	Biopac MP150 (BIOPAC Hardware)	Reaction time to peripheral stress inducing driving tasks	ML: K-Nearest Neighbour, Nearest Mean Classifier, Multilayer perceptron	Machine learning classification algorithms (MLP, K-NN) classified uncorrelated features to achieve 91% accuracy.
[75]	HR, HRV	On-road	Zephyr BioHarness 3 (Zephyr technology)	Secondary task interaction with in-vehicle infotainment system	Repeated measure ANOVA	Increase in HR from single-task to dual-task condition.
[59]	HR, HRV, RR	Simulator	G-Tech medical sensors (G-Tech Medical Inc. Austria)	Traffic intensity	Pearson correlation	HRV features has high correlation with subjective feedback. Moderate correlation with breathing frequency.
[76]	HR, HRV	Simulator	Polar H7 heart monitor (Polar Electro Inc.)	Rural vs urban vs motorway scenario	Multivariate ANOVA, Post-hoc Pairwise comparison	Pairwise comparison showed RMSSD and pNN50 variance as insignificant between motorway and rural roads. VLF variance was insignificant between rest and rural scenarios.
[56]	HR, HRV, RR	Simulator	Portable Mobii ECG device, RespiV6 sensor (TMS International BV)	Time-pressure vs non time-pressure scenario	Paired *t*-tests	Increased HR, RR in time-pressure scenario. Task needs to be constant with time to interpret HRV features.
[60]	HR, HRV	On-road	ErgoLAB ECG sensors (Grennlee ErgoLAB)	Intersection turning behaviour	SDNN and RMSSD comparison	SDNN and RMSSD was higher in turning scenarios. No changes in pNN50 were observed.
[61]	HR, HRV, RR	Simulator	Biopac MP100 (BIOPAC Hardware)	Critical driving scenario	Multivariate ANOVA, Post-hoc analysis with univariate measures	Increase in HR in dual-task conditions. No significant changes in RR.
[62]	HR, HRV	Simulator	MEDAC ECG System (MEDAC Tech Co., Ltd.)	N-back tasks	One-factor within subject ANOVA, Artificial Neural Network classification	Time domain measures were more sensitive (IBI, RMSSD, SDNN) and decreased with increase in task difficulty.
[52]	HR, HRV	On-road	Electrocardiograph 300 G (Bionex Medical Equipemnt)	City vs rural vs motorway roads	Relationship between mean HR and frustration factor subjective data	High correlation between subjective and R-R interval. Least mean RR was observed on city roads, high mean RR was observed on motorways.
[54]	HR, HRV	On-road	Eight-slot Bionex ECG 5003711-08 (Bionex Medical Equipment)	Drive by speed signs	Repeated measures ANOVA	One cluster of participants responded strongly to intensive braking, increasing HR and decreasing mid-band HR. Task similarity affects MWL.
[79]	HR, HRV	On-road	Optical sensor (HR)—Atmel AtMega328 P (Microchip Technology)	Traffic density, effect of autopilot feature, occupant interaction	ANOVA	HR and HRV features did not show any significant effects from environmental changes or use of the autopilot feature. Subjective ratings suggest reduced workload with automation in automation-experienced drivers.
[63]	HR, HRV, RR	On-road	MLA2505 biopotential ECG, MLT1132 respiratory belt (ADInstruments)	Lane changing behaviour	No statistical tests as smaller sample size	HR decreases with time on task. The LF/HF features of HRV were affected by driver posture, vehicle vibration and did not yield the same results as observed in subjective feedback.
[64]	HR, HRV	On-road	Biopac—Bionomadix (BIOPAC Hardware)	Impact of lane keeping assisting system (LKAS)	ANOVA with repeated measures, Post-hoc pairwise comparison	No major impact of LKAS on HR, HRV. HR was higher on curvy road when compared to straight motorway.
[65]	HR, HRV	Simulator	Biopac MP36R (BIOPAC Hardware)	Types of music	Mixed ANOVA	Mean HR difference was noticed in sanguine drivers with rock music. Phlegmatic drivers showed low arousal levels in general and has high tolerance to stimulus. No significant difference for melancholic drivers. Driving impairment was observed in choleric drivers.
[82]	HR, HRV	Simulator	Android smartwatch-PPG (Manufacturer not specified)	Weather conditions, road type, passenger presence	Comparison of RMSSD	RMSSD is used as a dependent variable. HRV increases in highways compared to city roads. Stress levels decreased in the presence of a passenger.
[66]	HR, HRV	Simulator	Biopac MP150 (BIOPAC Hardware)	Effect of fatigue, gender-related differences	Mann-Whitney U tests	HRV time and frequency features domain tend to show significant difference between alert and fatigued states. HRV time and frequency domain features had gender differences in detecting mental workload.
[77]	HR, HRV	Simulator	Polar H10 heart monitor (Polar Electro Inc.)	Types of Music	Mixed-model condition x personality (M)ANOVA, Pairwise comparisons	Mean HR did not reveal any significant differences to different auditory input.
[57]	HR, HRV	Simulator	Polar H10 heart monitor (Polar Electro Inc.)	N-back tasks	One-way ANOVA	HR was found to be higher in urban and motorway scenarios. Time domain measures of HR and HRV seemed to be more sensitive to changes in MWL.
[81]	HR, HRV	On-road	Ear lobe PPG-based sensor (Manufacturer not specified)	Road geometry	ANOVA, ML: K-Means clustering	An increase in visual input increased HR. The effect of geometry on road is not very different among participants of different age, occupation, driving experience or reaction time.
[67]	HR, HRV	Simulator	PhysioLab wireless ECG device (PhysioLav Co., Ltd)	Illumination, longitudinal road slope	Two-way repeated measures ANOVA	Inconsistent results were observed in HRV time domain features due to changes in luminance and road slope.
[68]	HR, HRV	Simulator	Biopac MP160 BioNomadix (BIOPAC Hardware)	Traffic density, weather conditions, speed, highway vs city vs dual carriage roads	Parametric statistical analysis (T-scores)	Highway—increased HR and HF/LF ratio Interurban—increased HR and lower RMSSD Urban low-complexity—increased HR, LF/HF ratio, reduced RMSSD
[69]	HR, HRV	Simulator	Encephalan Mini (Medicom MTD system, Taganrog, Russia)	Short-term vs long-term verbal learning	ML: Decision Trees, Discriminant Analysis, Logistic regression, Support Vector Machines, Nearest Neighbour, Ensemble classifiers	HF/LF features of HRV showed significant changes only for verbal and auditory learning tests. The best classification accuracies were achieved with multimodal IR + HRV features.
[78]	HR, HRV	Simulator	Polar H10 heart monitor (Polar Electro Inc.)	Secondary task (In-vehicle interference)	One-sample two-sided *t*-tests	Significant effect of task difficulty on HR, the lower difference in HR in task conditions compared to no task conditions. Significant changes in the number of successes in task performance.
[80]	HR, HRV	Simulator	Smartwatch-PPG (Manufacturer not specified)	Traffic density, stress levels	Log-likelihood	Effects of traffic density on mental workload showed individual differences between participants. The model shows that stress and workload are dependent on historic values.
[53]	HR, HRV	On-road	Likon Prince180D heart rate tester (North-vision Tech. Inc.)	Optical vs non-optical tunnels	SDNN and RMSSD comparison	Increased mean HR when driving in tunnels compared to resting state, no significant difference between optical and non-optical tunnels. Non-optical tunnel SDNN was lesser than optical tunnel SDNN. RMDDS decreased in non-optical tunnels compared to optical tunnels.
[30]	HR, HRV, RR	Simulator	Biopac MP150 BioNomadix (BIOPAC Hardware)	Low cognitive vs high cognitive mental arithmetic secondary task	Two-way ANOVA	HR increased for high cognitive tasks. RMSSD decreased, showing opposing effects. pNN20 decreased. No significant results for SDNN. RR increased for the driving condition, with no change in a high cognitive task.
[70]	HR, HRV, RR	Simulator	Biopac MP36 (BIOPAC Hardware)	Verbal cognitive secondary tasks	ANOVA	ECG + RR signals presented the best accuracy for the classifier (92–94%).
[6]	HR, HRV	Simulator	PolymateV AP5148 (Miyuki Giken, AnalyzeDirect Inc.)	Traffic density, pedestrians	ML: Long Short-Term Memory classification	LSTM-based 5-class classification was performed to achieve 96.5% accuracy in relation with subjective measures.
[71]	HR, HRV, RR	On-road	3-lead ECG, respiratory belt (Manufacturer not specified)	N-back tasks	ANOVA, Paired t-test, ML: CNN-LSTM, CNN, Conv-LSTM, XGBoost	CNN and LSTM had the best classification results (97.8%). Four classes: relaxed, normal, high, very high.
[72]	HR, HRV	Simulator	Microvit MT 101 (Schiller)	Lane changing task, secondary N-back task	ANOVA, Post-hoc pairwise comparison	RMSSD, SDSD, pNN20, pNN50, and HF remained constant from rest to level 1 and increased from level 1 to 2. Only three parameters showed difference between levels 2 and 3: LF, HF, pNN50.
[73]	HR, HRV	Simulator	Biopac MP36 (BIOPAC Hardware)	Overtaking events, pedestrians	ANOVA, ML: Random Forest, C-support vector classification, multilayer perceptron	Considering short-term windows, HR and HRV increased in overtaking events and in the presence of pedestrians.

## Data Availability

Not applicable.
